# Association between FIASMA psychotropic medications and reduced risk of intubation or death in individuals with psychiatric disorders hospitalized for severe COVID-19: an observational multicenter study

**DOI:** 10.1038/s41398-022-01804-5

**Published:** 2022-03-03

**Authors:** Nicolas Hoertel, Marina Sánchez-Rico, Erich Gulbins, Johannes Kornhuber, Alexander Carpinteiro, Miriam Abellán, Pedro de la Muela, Raphaël Vernet, Nathanaël Beeker, Antoine Neuraz, Aude Delcuze, Jesús M. Alvarado, Céline Cougoule, Pierre Meneton, Frédéric Limosin, On behalf of AP-HP/Université de Paris/INSERM COVID-19 research collaboration/AP-HP COVID CDR Initiative/“Entrepôt de Données de Santé” AP-HP Consortium

**Affiliations:** 1 AP-HP, Hôpital Corentin-Celton, DMU Psychiatrie et Addictologie, Département de Psychiatrie, Issy-les-Moulineaux, France; 2 Université de Paris, Paris, France; 3 INSERM, Institut de Psychiatrie et Neurosciences de Paris (IPNP), UMR S1266 Paris, France; 4 Universidad Complutense de Madrid, Department of Psychobiology & Behavioural Sciences Methods, Faculty of Psychology, Campus de Somosaguas, Pozuelo de Alarcon, Spain; 5 Institute for Molecular Biology, University Medicine Essen, University of Duisburg- Essen, Essen, Germany; 6 Department of Psychiatry and Psychotherapy, University Hospital, Friedrich- Alexander-University of Erlangen-Nuremberg, Erlangen, Germany; 7 Department of Hematology and Stem Cell Transplantation, University Hospital Essen, University of Duisburg-Essen, Essen, Germany; 8 AP-HP.Centre-Université de Paris, Hôpital Européen Georges Pompidou, Medical Informatics, Biostatistics and Public Health Department, Paris, France; 9 Assistance Publique-Hopitaux de Paris, Unité de Recherche clinique, Hopital Cochin, Paris, France; 10 INSERM, UMR S1138, Cordeliers Research Center, Université de Paris, Paris, France; 11 AP-HP.Centre-Université de Paris, Department of Medical Informatics, Necker-Enfants Malades Hospital, 75015 Paris, France; 12 ORPEA - CLINEA, Clinique Les Orchidées, Service de Psychiatrie, Andilly, France; 13 Institut de Pharmacologie et de Biologie Structurale, IPBS, Université de Toulouse, Toulouse, France; 14 INSERM U1142 LIMICS, UMR S1142, Sorbonne Universities, UPMC University of Paris 06, University of Paris 13, Paris, France

**Keywords:** Psychiatric disorders, Pathogenesis

## Abstract

The acid sphingomyelinase (ASM)/ceramide system may provide a useful framework for better understanding SARS-CoV-2 infection and the repurposing of psychotropic medications functionally inhibiting the acid sphingomyelinase/ceramide system (named FIASMA psychotropic medications) against COVID-19. We examined the potential usefulness of FIASMA psychotropic medications in patients with psychiatric disorders hospitalized for severe COVID-19, in an observational multicenter study conducted at Greater Paris University hospitals. Of 545 adult inpatients, 164 (30.1%) received a FIASMA psychotropic medication upon hospital admission for COVID-19. We compared the composite endpoint of intubation or death between patients who received a psychotropic FIASMA medication at baseline and those who did not in time-to-event analyses adjusted for sociodemographic characteristics, psychiatric and other medical comorbidity, and other medications. FIASMA psychotropic medication use at baseline was significantly associated with reduced risk of intubation or death in both crude (HR = 0.42; 95%CI = 0.31–0.57; *p* < 0.01) and primary inverse probability weighting (IPW) (HR = 0.50; 95%CI = 0.37–0.67; *p* < 0.01) analyses. This association was not specific to one FIASMA psychotropic class or medication. Patients taking a FIASMA antidepressant at baseline had a significantly reduced risk of intubation or death compared with those taking a non-FIASMA antidepressant at baseline in both crude (HR = 0.57; 95%CI = 0.38–0.86; *p* < 0.01) and primary IPW (HR = 0.57; 95%CI = 0.37–0.87; *p* < 0.01) analyses. These associations remained significant in multiple sensitivity analyses. Our results show the potential importance of the ASM/ceramide system framework in COVID-19 and support the continuation of FIASMA psychotropic medications in these patients and the need of large- scale clinical trials evaluating FIASMA medications, and particularly FIASMA antidepressants, against COVID-19.

## Introduction

Global spread of the novel coronavirus SARS-CoV-2, the causative agent of coronavirus disease 2019 (COVID-19), has created an unprecedented infectious disease crisis worldwide [1–5]. Although the availability of vaccines has raised hope for a decline of the pandemic, the search for an effective treatment for patients with COVID-19 is still urgently needed, especially those easy to use, including good tolerability, oral administration, widespread availability, and low cost, to allow their use in resource-poor unvaccinated countries [6, 7].

Prior preclinical evidence supports that SARS-CoV-2 activates the acid sphingomyelinase (ASM)/ceramide system, resulting in the formation of ceramide-enriched membrane domains that serve viral entry and infection by clustering ACE2, the cellular receptor of SARS-CoV-2 [8–10]. An in vitro study [8] showed that several FIASMA (Functional Inhibitors of Acid Sphingomyelinase Activity) [11] antidepressant medications, including amitriptyline, imipramine, desipramine, fluoxetine, sertraline, escitalopram, and maprotiline, inhibited ASM and the formation of ceramide-enriched membrane domains and prevented Vero E6 cells from being infected with SARS-CoV-2. Importantly, reconstitution of ceramides in cells treated with these FIASMA antidepressants restored infection with SARS-CoV-2. Oral administration of the FIASMA antidepressant amitriptyline in healthy volunteers also efficiently blocked infection of freshly isolated nasal epithelial cells with SARS-CoV-2 [8]. These preclinical data were confirmed by another study that demonstrated an inhibition of the infection of cultured epithelial cells with SARS-CoV-2 by the FIASMA antidepressant fluoxetine [12]. Furthermore, a retrospective cohort study of an adult psychiatric facility operated by the New York State Office of Mental Health found a significant and substantial protective association between the use of antidepressants, many of them being FIASMA, and COVID-19 infection [13]. Finally, plasma levels of ceramides were found to strongly correlate with disease clinical severity [14–16] and with inflammation markers [14, 16] in patients with COVID-19.

From a theoretical perspective, functional inhibition of ASM requires only a few structural conditions: the molecules need to contain a lipophilic organic ring that integrates into the inner lysosomal membrane, a short spacer and a charged tertiary amine group that displaces ASM from the inner lysosomal membrane, which results in the proteolysis of the enzyme in the lysosomal lumen [9, 17, 18]. All FIASMAs identified so far include mono-, bi-, tri- and tetracyclic compounds and have at least one basic nitrogen atom, have a medium to high logP value, and most of them have a molecular weight below 500. FIASMAs more frequently violate Lipinski’s Rule-of-Five than non-FIASMA compounds and appear to have good permeability across the blood−brain barrier [11]. Conversely, not all lipophilic weak bases are FIASMAs [11]. Based on these molecular properties and their in vitro functional inhibition effect on ASM (i.e., a residual ASM activity lower than 50%), psychotropic medications can be subdivided into FIASMA psychotropic medications (e.g., fluoxetine, fluvoxamine, escitalopram, aripiprazole, hydroxyzine) and non-FIASMA psychotropic medications (e.g., mianserin, haloperidol, lamotrigine, donepezil). FIASMA psychotropic medications include certain antidepressants (i.e., amitriptyline, clomipramine, desipramine, doxepin, duloxetine, escitalopram, fluoxetine, fluvoxamine, imipramine, lofepramine, maprotiline, nortriptyline, paroxetine, protriptyline, sertraline, and trimipramine), certain antipsychotics (i.e., aripiprazole, chlorpromazine, chlorprothixene, fluphenazine, flupenthixol, penfluridol, perphenazine, pimozide, promazine, sertindole, thioridazin, trifluoperazine, and triflupromazine), and hydroxyzine [8–11, 19, 20].

The potential benefit of FIASMA medications in patients hospitalized for severe COVID-19 was recently explored in an observational multicenter retrospective study using data from the Assistance Publique-Hôpitaux de Paris (AP-HP) Health Data Warehouse [21]. Therein, it was reported that taking a FIASMA medication upon hospital admission was significantly associated with substantially reduced likelihood of intubation or death. A retrospective observational study also established a similar association between chronic administration of a FIASMA medication and diminished mortality in patients hospitalized with COVID-19, that was significant for the FIASMA amlodipine [22]. Furthermore, data from the AP-HP Health Data Warehouse [23–25] showed that use of antidepressants, many of them being FIASMA, and the FIASMA hydroxyzine, were significantly associated with reduced mortality in patients hospitalized for COVID-19. Finally, results from a large US retrospective cohort study found that selective serotonin reuptake inhibitor (SSRI) antidepressants, and more specifically fluoxetine and fluoxetine or fluvoxamine, which are FIASMA, were significantly associated with reduced mortality [26]. Specifically, fluoxetine or fluvoxamine were significantly associated with reduced risk of death (48 of 481 [10.0%] vs 956 of 7215 [13.3%]; RR, 0.74 [95% CI, 0.55–0.99]; adjusted *p* = 0.04), while the association between receiving any SSRI that were not fluoxetine and fluvoxamine, and reduced mortality showed a non-significant trend (447 of 2898 [15.4%] vs 1474 of 8694 [17.0%]; RR, 0.92 [95% CI, 0.84–1.00]; adjusted *p* = 0.06) [26]. However, results from these observational studies might be biased due to possible confounding by indication or unmeasured confounding [25]. Examining this potential association among a more homogeneous population sharing the main medical indications linked to the prescription of the studied medications (e.g., individuals with psychiatric disorders and SARS-CoV-2 infection for studying the potential effects of psychotropic medications on COVID-19 disease severity) may help reduce these biases.

Findings from four clinical trials are consistent with a potential benefit of FIASMA medications in patient with COVID-19. First, a randomized double-blind controlled study [27] showed significant and substantial protective effects of the FIASMA antidepressant fluvoxamine (*N* = 80) versus placebo (*N* = 72) on COVID-19 disease progression in outpatients. Second, the results of a prospective real-world evidence study [28] including 113 outpatients with COVID- 19 also support this observation. Third, the results of the multi-center randomized placebo-controlled TOGETHER trial performed by Reis, Mills, and colleagues showed a significant and substantial reduction in risk of hospitalization or retention in a COVID-19 emergency setting due to COVID-19 associated with fluvoxamine use versus placebo in 1,497 outpatients with COVID-19 at a high risk for severe complications [29]. Finally, an open-label prospective cohort trial with matched controls involving 102 hospitalized ICU COVID-19 patients reported that fluvoxamine treatment in addition to the standard therapy was significantly and substantially associated with reduced mortality [30].

Taken together, these preclinical, observational, and clinical findings suggest that the ASM/ceramide system may provide a useful framework for better understanding SARS-CoV-2 infection [9], the antiviral and anti-inflammatory effects of functional inhibitors of the ASM [9], and the repurposing of FIASMA psychotropic medications [6, 7], whose short-term use is generally well-tolerated [31–33], against COVID-19.

To our knowledge, no clinical study to date has examined the potential usefulness of FIASMA psychotropic medications in patients with psychiatric disorders hospitalized for COVID-19. Observational studies of patients with COVID-19 taking medications for other indications can help decide which treatment should be prioritized for randomized clinical trials and reduce the risk for patients of being exposed to potentially harmful and ineffective treatments [23, 24, 34–36]. Observational studies examining the potential usefulness of FIASMA psychotropic medications in COVID-19 are also important because in vitro FIASMA effects may not translate to clinical FIASMA effects in humans for all individual FIASMA psychotropic medications, which depends on the dose used and pharmacokinetics of each molecule. For example, a prior observational study showed that use of the FIASMA chlorpromazine prescribed at a mean daily dose of 70.8 mg (SD = 65.3) in 55 adult patients hospitalized for COVID-19 was not significantly associated with mortality in COVID-19 [35]. However, beyond the issue of limited statistical power, we cannot rule out that the clinical FIASMA properties of chlorpromazine might be observable at higher doses, especially given that the in vitro FIASMA effect of chlorpromazine is rather low (residual ASM activity of 42%) [20]. Therefore, observational studies focused on the potential usefulness of a biological mechanism shared by several existing treatments in COVID-19 can be very useful to help guide the choice of the molecules to prioritize in clinical trials.

In this report, we used data from the AP-HP Health Data Warehouse and examined the association between FIASMA psychotropic medication use and the composite outcome of intubation or death among patients with psychiatric disorders hospitalized for severe COVID-19. We focused on this population because individuals with psychiatric disorders are at higher risk of severe COVID-19 [23, 37, 38] and are likely to receive psychotropic medications for treating or preventing the relapse of psychiatric disorders. Furthermore, the choice of this population aimed at reducing the risk of confounding by indication. If a significant protective association was found, we sought to perform additional exploratory analyses to examine whether this association was specific to certain FIASMA psychotropic classes (e.g., antidepressants) or individual medications (e.g., fluoxetine). Our primary hypothesis was that FIASMA psychotropic medication use would be associated with reduced risk of intubation or death in patients with psychiatric disorders hospitalized for severe COVID-19 in time-to-event analyses adjusting for sociodemographic characteristics, psychiatric and other medical comorbidity, and other medications. If it was the case, our secondary hypothesis was that this association would not be specific to one FIASMA psychotropic class or medication.

## Methods

### Setting and cohort assembly

A multicenter observational retrospective cohort study was conducted at 36 AP-HP hospitals from the beginning of the epidemic in France (i.e., January 24^th^, 2020) until May 1^st^, 2020 [21, 23, 34–36]. We included all adults aged 18 years or over with a psychiatric disorder who had been hospitalized in these medical centers for severe COVID-19. Psychiatric disorder was defined as having at least one current International Statistical Classification of Diseases and Related Health Problems (ICD-10) diagnosis of psychiatric disorder (F01-F99) during the visit or an ongoing prescription of any antidepressant, antipsychotic, or mood stabilizer (i.e. lithium or antiepileptic medications with mood stabilizing effects) at hospital admission. COVID-19 was ascertained by a positive reverse-transcriptase–polymerase-chain-reaction (RT-PCR) test from analysis of nasopharyngeal or oropharyngeal swab specimens. Severe COVID-19 was defined as having at least one of the following criteria at baseline [39–41]: respiratory rate >24 breaths/min or <12 breaths/min, resting peripheral capillary oxygen saturation in ambient air <90%, temperature >40 °C, systolic blood pressure <100 mm Hg, lactate levels >2 mmol/L, or admission to an intensive care unit (ICU) within the first 24 h form hospital admission.

This observational study using routinely collected data received approval from the Institutional Review Board of the AP-HP clinical data warehouse (decision CSE-20–20_COVID19, IRB00011591). AP-HP clinical Data Warehouse initiatives ensure patient information and informed consent regarding the different approved studies through a transparency portal in accordance with European Regulation on data protection and authorization n°1980120 from National Commission for Information Technology and Civil Liberties (CNIL).

### Data sources

We used data from the AP-HP Health Data Warehouse (‘Entrepôt de Données de Santé (EDS)’). This warehouse contains all available clinical data on all inpatient visits for COVID-19 to 36 Greater Paris University hospitals. The data included patient demographic characteristics, vital signs, laboratory test and RT-PCR test results, medication administration data, medication lists during current and past hospitalizations in AP-HP hospitals, current diagnoses, discharge disposition, ventilator use data, and death certificates.

### Variables assessed

We obtained the following data for each patient at the time of the hospitalization: sex; age; hospital; obesity; current smoking status; any medication prescribed according to compassionate use or as part of a clinical trial; current psychiatric disorder (i.e. ICD-10 diagnosis of substance use disorder, psychotic disorder, mood or anxiety disorder, delirium or dementia, and other psychiatric disorders); and any prescription for antidepressant, mood stabilizer, benzodiazepine or Z-drug, or antipsychotic medication. These variables are detailed in Supplementary Text.

### Psychotropic medications functionally inhibiting the acid sphingomyelinase (ASM)/ceramide system (or FIASMA psychotropic medications)

FIASMA psychotropic medications were defined as psychotropic medications showing a substantial in vitro functional inhibition effect on ASM (i.e., a residual ASM activity lower than 50%), as described in detail elsewhere [8–11, 19, 20]. If there were no experimental data from our group regarding FIASMA activity for an administered psychotropic medication, these substances were assigned to non-FIASMA psychotropic medication. FIASMA psychotropic medication use was defined as receiving at least one psychotropic FIASMA medication at study baseline, i.e., within the first 24 h of hospital admission, and before the end of the index hospitalization, intubation or death. To minimize potential confounding effects of late prescription of FIASMA psychotropic medications, patients who received a FIASMA psychotropic medication more than 24 h after hospital admission were excluded from the analyses. Finally, patients who received at study baseline an antipsychotic while being hospitalized in an ICU, possibly as an aid to oral intubation, were also excluded.

### Primary endpoint

Study baseline was defined as the date of hospital admission for COVID-19. The primary endpoint was the occurrence of intubation and/or death. For patients who died after intubation, the timing of the primary endpoint was defined as the time of intubation. Patients without an end-point event had their data censored on May 1^st^, 2020.

### Statistical analysis

We calculated frequencies of baseline characteristics described above in patients receiving or not receiving a FIASMA psychotropic medication and compared them using standardized mean differences (SMD).

To examine the association between FIASMA psychotropic medication use upon hospital admission and the endpoint of intubation or death, we performed Cox proportional- hazards regression models [42]. To help account for the nonrandomized prescription of psychotropic medications and reduce the effects of confounding, the primary analysis used propensity score analysis with inverse probability weighting (IPW) [43, 44]. Given the expected relatively limited sample size and the number of potentially influencing variables, a backward stepwise Cox regression was used to assess the importance of the covariates (listed in Supplementary Table 1) on the outcome, based on clinical meaningfulness and the Akaike Information Criterion (AIC) for model comparison [45]. Next, the individual propensities for receiving a FIASMA psychotropic medication at baseline were estimated using a multivariable logistic regression model including the variables from the model with the lowest AIC value. In the inverse-probability-weighted analyses, the predicted probabilities from the propensity-score models were used to calculate the stabilized inverse-probability-weighting weights [43]. The association between FIASMA psychotropic medication use and the endpoint was then estimated using an IPW Cox regression model. In case of unbalanced covariates, an IPW multivariable Cox regression model adjusting for the unbalanced covariates was also performed. Kaplan-Meier curves were performed using the inverse-probability-weighting weights [45, 46] and their pointwise 95% confidence intervals were estimated using the nonparametric bootstrap method [47].

We conducted two sensitivity analyses. First, we performed a multivariable Cox regression model including as covariates the same variables used in the IPW analysis. Second, we used a univariate Cox regression model in a matched analytic sample using a 1:1 ratio, based on the same variables used for the IPW analysis and the multivariable Cox regression analysis. To reduce the effects of confounding, optimal matching was used in order to obtain the smallest average absolute distance across all clinical characteristics between exposed patients and non-exposed matched controls [48].

We performed four additional exploratory analyses. First, we examined the relationships between each FIASMA psychotropic class (i.e. FIASMA antidepressants and antipsychotics) and each individual FIASMA molecule with the endpoint. Second, we examined within each psychotropic class (i.e. antidepressants and antipsychotics) the relationships of FIASMA and non-FIASMA molecules with the endpoint. Third, because of discrepancies in the potential FIASMA in vitro effect of venlafaxine, mirtazapine, and citalopram [8, 11], we reproduced the main analyses while considering these molecules as FIASMAs. Finally, we reproduced the main analyses among all patients with psychiatric disorders with and without clinical severity criteria at baseline. In exploratory analyses, the matched analytic samples were built using a 1:2 ratio (2 controls per case).

For all associations, we performed residual analyses to assess the fit of the data, checked assumptions, including proportional hazards assumption using proportional hazards tests and diagnostics based on weighted residuals [42, 49], and examined the potential influence of outliers. Because our main analysis focused on the association between FIASMA psychotropic medication use and the composite outcome of intubation or death among patients with psychiatric disorders hospitalized for severe COVID-19, statistical significance was fixed a priori at two-sided *p*-value < 0.05. Only if a significant protective association was found, we planned to perform additional exploratory analyses as described above. All analyses were conducted in R software version 2.4.3 (R Project for Statistical Computing).

## Results

### Characteristics of the cohort

**Fig. 1:**
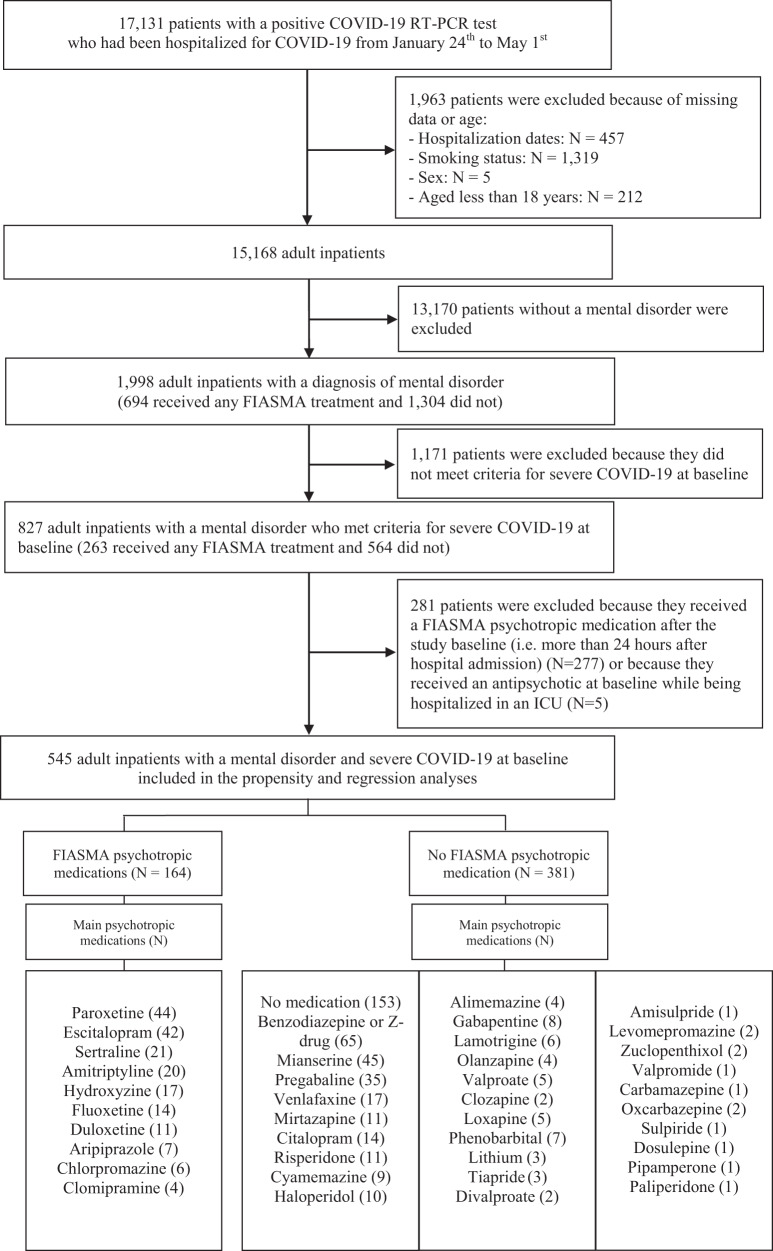
Study cohort.

Of the 17,131 patients with a positive COVID-19 RT-PCR test who had been hospitalized for COVID-19, 1963 (11.5%) were excluded because of missing data or young age (i.e., less than 18 years old of age). Of 15,168 adult inpatients, 1998 (13.2%) had a psychiatric disorder diagnosis or an ongoing prescription of any antidepressant, antipsychotic, or mood stabilizer at hospital admission. Of these 1,998 patients, 827 (41.4%) had criteria for severe COVID-19. Of these 827 patients, 281 (34.0%) were excluded because they received a FIASMA psychotropic medication more than 24 h from hospital admission (*N* = 277) or because they initiated an antipsychotic in an ICU, possibly as an aid for intubation (*N* = 5). Of the remaining 545 adult inpatients with psychiatric disorders and severe COVID-19, 164 (30.1%) received a FIASMA psychotropic medication at baseline and 381 (69.9%) did not (Fig. 1).

Over a mean follow-up of 9.2 days (SD = 12.5; median = 6 days), 272 patients (50.0%) had an end-point event at the time of data cutoff on May 1st, 2020. Among patients who received a FIASMA psychotropic medication at baseline, the mean follow-up was 12.0 days (SD = 12.9, median=8 days), while it was of 8.9 days (SD = 12.4, median=5 days) in those who did not.

Sex, hospital, number of medical conditions, delirium or dementia, any other psychiatric disorder, and the prescription of any antidepressant, any antipsychotic, and any mood stabilizer were significantly associated with the endpoint of intubation or death (Supplementary Table 1). A backward stepwise Cox regression showed that a model including age, sex, hospital, obesity, and the number of medical conditions, was meaningful and associated with the lowest AIC value (Supplementary Table 2).

The distributions of patient characteristics included in the propensity and regression analyses according to FIASMA psychotropic medication use at baseline are shown in Table 1. In the full sample, FIASMA psychotropic medication use substantially differed according to age, sex, hospital, and number of medical conditions. After applying the propensity score weights, there were no substantial differences (i.e., all SMD < 0.1) in any characteristic. In the matched analytic sample using a 1:1 ratio, sex and the number of medical conditions differed between groups (Table 1).

**Table 1:** Characteristics of patients with psychiatric disorders and severe COVID-19 receiving or not receiving FIASMA psychotropic medications at baseline (*N* = 545).

	Exposed to any FIASMA (*N* = 164)	Not exposed to any FIASMA (*N* = 381)	Non-exposed matched group (*N* = 164)	Exposed to any FIASMA vs. Not exposed	Exposed to any FIASMA vs. Not exposed	Exposed to any FIASMA vs. Non-exposed matched group
				Crude analysis	Analysis weighted by inverse-probability- weighting weights	Matched analytic sample analysis
	*N* (%)	*N* (%)	*N* (%)	SMD	SMD	SMD
Age				0.197	0.037	0.022
18 to 50 years	19 (11.6%)	26 (6.82%)	18 (48.6%)			
51 to 70 years	43 (26.2%)	105 (27.6%)	43 (50.0%)			
71 to 80 years	37 (22.6%)	75 (19.7%)	38 (50.7%)			
More than 80 years	65 (39.6%)	175 (45.9%)	65 (50.0%)			
Sex				**0.260**	0.008	**0.159**
Women	90 (54.9%)	160 (42.0%)	77 (46.1%)			
Men	74 (45.1%)	221 (58.0%)	87 (54.0%)			
Hospital				**0.267**	0.024	0.068
AP-HP Centre – Paris University, Henri Mondor University Hospitals and at home hospitalization	36 (22.0%)	115 (30.2%)	40 (52.6%)			
AP-HP Nord and Hôpitaux Universitaires Paris Seine-Saint-Denis	45 (27.4%)	77 (20.2%)	46 (50.5%)			
AP-HP Paris Saclay University	41 (25.0%)	113 (29.7%)	38 (48.1%)			
AP-HP Sorbonne University	42 (25.6%)	76 (19.9%)	40 (48.8%)			
Obesity^a^				**0.129**	0.001	**0.162**
Yes	42 (25.6%)	77 (20.2%)	31 (42.5%)			
No	122 (74.4%)	304 (79.8%)	133 (52.2%)			
Number of medical conditions^b^				**0.321**	0.015	0.029
0	42 (25.6%)	51 (13.4%)	40 (48.8%)			
1	17 (10.4%)	37 (9.71%)	17 (50.0%)			
2 or more	105 (64.0%)	293 (76.9%)	107 (50.5%)			

^a^ Defined as having a body-mass index higher than 30 kg/m^2^ or an International Statistical Classification of Diseases and Related Health Problems (ICD-10) diagnosis code for obesity (E66.0, E66.1, E66.2, E66.8, E66.9).

^b^ Assessed using ICD-10 diagnosis codes for diabetes mellitus (E11), diseases of the circulatory system (I00-I99), diseases of the respiratory system (J00-J99), neoplasms (C00-D49), diseases of the blood and blood-forming organs and certain disorders involving the immune mechanism (D5-D8), frontotemporal dementia (G31.0), peptic ulcer (K27), diseases of liver (K70-K95), hemiplegia or paraplegia (G81-G82), acute kidney failure or chronic kidney disease (N17-N19), and HIV (B20).

SMD > 0.1 in bold indicate substantial differences.

*SMD* standardized mean difference.

### Study endpoint

**Fig. 2:**
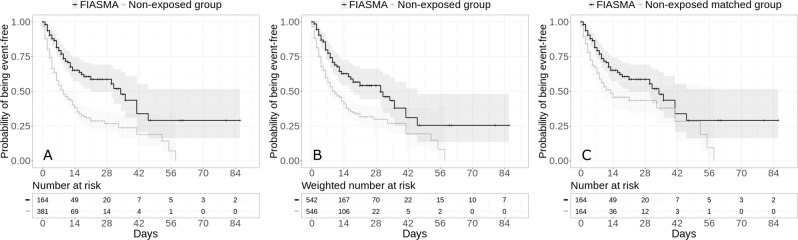
Kaplan-Meier curves for the composite endpoint of intubation or death among patients with psychiatric disorders hospitalized for severe COVID-19, according to FIASMA psychotropic medication use at baseline. Kaplan–Meier curves for the composite endpoint of intubation or death in the full sample crude analysis (*N* = 545) (**A**), in the full sample analysis with IPW (*N* = 545) (**B**), and in the matched analytic sample using a 1:1 ratio (*N* = 328) (**C**) among patients with psychiatric disorders hospitalized for severe COVID-19, according to FIASMA psychotropic medication use at baseline.

The endpoint of intubation or death occurred in 57 patients (34.8%) who received a FIASMA psychotropic medication at baseline and 215 patients (56.4%) who did not. The crude, unadjusted analysis (hazard ratio (HR) = 0.42; 95% CI = 0.31–0.57; *p* < 0.001) and the primary analysis with inverse probability weighting (HR = 0.50; 95% CI = 0.37–0.67; *p* < 0.001) showed a significant and substantial association between FIASMA psychotropic medication use at baseline and reduced risk of intubation or death (Fig. 2; Table 2). A post-hoc analysis indicated that we had 80% power in the crude analysis to detect a hazard ratio of at least 0.60 / 1.71.

**Table 2:** Association between FIASMA psychotropic medication use at baseline and risk of intubation or death among patients with psychiatric disorders hospitalized for severe COVID-19.

	Number of events / Number of patients	Crude Cox regression analysis	Multivariable Cox regression analysis^a^	Analysis weighted by inverse- probability- weighting weights^a^	Number of events / Number of patients in the matched groups	Univariate Cox regression in a 1:1 ratio matched analytic sample	Cox regression in a 1:1 ratio matched analytic sample adjusted for unbalanced covariates^b^
	*N* (%)	HR (95% CI; *p*-value)	HR (95% CI; *p*-value)	HR (95% CI; *p*-value)	*N* (%)	HR (95% CI; *p*-value)	HR (95% CI; *p*-value)
No FIASMA psychotropic medication	215 / 381 (56.4%)	Ref.	Ref.	Ref.	77 / 164 (47%)	Ref.	Ref.
Any FIASMA psychotropic medication	57 / 164 (34.8%)	0.42 (0.31–0.57; <0.001*)	0.49 (0.36–0.67; <0.001*)	0.50 (0.37–0.67; <0.001*)	57 / 164 (34.8%)	0.65 (0.45–0.93; 0.019*)	0.55 (0.39–0.77; 0.001*)

^a^Adjusted for age, sex, hospital, obesity, and number of medical conditions.

^b^Adjusted for sex and obesity.

^*^Two-sided *p*-value is significant (*p* < 0.05).

*HR* hazard ratio, *CI* confidence interval.

Non-FIASMA psychotropic medications include medications that have either no or a relatively low in vitro FIASMA effect corresponding to an in vitro residual ASM activity lower than 50%, or for which the FIASMA effect status is unknown.

In sensitivity analyses, the multivariable Cox regression model yielded a similar significant association (HR = 0.49; 95% CI = 0.36–0.67; *p* < 0.001), as did the Cox regression model in a matched analytic sample using a 1:1 ratio adjusted for unbalanced covariates, i.e., sex and number of medical conditions (HR = 0.55; 95% CI = 0.39–0.77; *p* = 0.001) (Table 2).

**Fig. 3:**
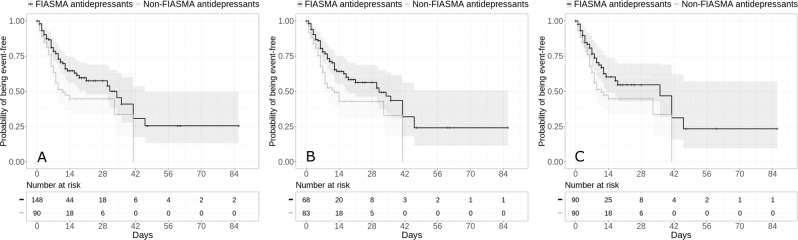
Kaplan-Meier curves for the composite endpoint of intubation or death among patients with psychiatric disorders hospitalized for severe COVID-19 and receiving a FIASMA versus a non-FIASMA antidepressant at baseline. Kaplan–Meier curves for the composite endpoint of intubation or death in the full sample crude analysis (*N* = 238) (**A**), in the full sample analysis with IPW (*N* = 238) (**B**), and in the matched analytic sample using a 1:1 ratio (*N* = 180) (**C**) among patients with psychiatric disorders hospitalized for severe COVID-19 and receiving a FIASMA *versus* a non-FIASMA antidepressant at baseline.

Additional exploratory analyses showed that FIASMA antidepressant use at baseline was significantly associated with reduced risk of intubation or death across all analyses (Supplementary Table 3). FIASMA antipsychotic use at baseline was significantly associated with reduced risk of intubation or death only in the multivariable Cox regression model and in the Cox regression model in a matched analytic sample using a 1:2 ratio adjusted for unbalanced covariates, possibly because of limited statistical power due to the limited number of patients receiving a FIASMA antipsychotic at hospital admission (*N* = 13) (Supplementary Table 3). Hazard ratios were lower than 1 for most individual FIASMA molecules, but none of them reached statistical significance across all main and sensitivity analyses, except for hydroxyzine and escitalopram, possibly because of limited statistical power due to individual sample sizes ≤44 patients. Patients receiving a FIASMA antidepressant at baseline (*N* = 148) had a significantly reduced risk of intubation or death compared with those receiving a non-FIASMA antidepressant at baseline (*N* = 90) (Supplementary Table 4; Fig. 3). Multivariable analyses comparing FIASMA and non-FIASMA antipsychotics and antihistaminic medications could not be performed due to the insufficient number of events (i.e., <5) in the FIASMA groups (Supplementary Table 4). Finally, reproducing the main analyses among all patients with psychiatric disorders with and without clinical severity criteria at baseline did not alter the significance of our results (Supplementary Table 5), as did the main analyses considering venlafaxine, mirtazapine and citalopram as FIASMA antidepressants (Supplementary Table 6).

## Discussion

In this multicenter retrospective observational study involving 545 adult patients with psychiatric disorders hospitalized for severe COVID-19 (*N* = 545), we found that FIASMA psychotropic medication use at study baseline was significantly and substantially associated with reduced risk of intubation or death, independently of sociodemographic characteristics, psychiatric and other medical comorbidity, and other medications. This association remained significant in multiple sensitivity analyses. Exploratory analyses suggested that this association was not specific to one FIASMA psychotropic class or medication in this population. They also indicate that patients taking a FIASMA antidepressant may have a significantly reduced risk of intubation or death compared with those taking a non-FIASMA antidepressant. These results suggest that the acid sphingomyelinase (ASM)/ceramide system may provide a useful framework for better understanding SARS-CoV-2 infection and the repurposing of FIASMA psychotropic medications, especially FIASMA antidepressants that are better tolerated than FIASMA antipsychotics [31–33, 50], against COVID-19 among individuals with psychiatric disorders. Our findings also support the urgent need of double-blind controlled randomized clinical trials of these medications for COVID-19 in this population, and especially FIASMA antidepressants such as fluoxetine, fluvoxamine or escitalopram, and more broadly in patients with severe COVID-19.

We found that FIASMA psychotropic medication use was significantly and substantially associated with reduced risk of intubation or death among patients with psychiatric disorders hospitalized for severe COVID-19, and that this association was not specific to one FIASMA psychotropic class or medication in this population. These findings are in line with prior preclinical [8, 12, 51–54], observational [21–24, 55–57], and clinical [27–30] evidence that FIASMA antidepressant medications may substantially prevent cells from being infected with SARS-CoV-2 in vitro [8, 12], and that FIASMA antidepressants and potentially the FIASMA anti-histamine hydroxyzine at their usual respective antidepressant and antihistaminic doses, may reduce mortality among patients with COVID-19.

As previously shown, functional inhibition of the ASM by FIASMA psychotropic medications results in lowering the amount of ceramide in the cell plasma membrane, thus preventing clustering of ACE2 in ceramide-enriched membrane domains and thereby protecting cells against infection with SARS-CoV-2 and reducing its spread and the virus-induced inflammation [8, 10]. Studies on biomarkers for COVID-19 demonstrated that blood plasma levels of ceramides were found to strongly correlate with disease clinical severity [14–16] and with inflammation markers in patients with COVID-19 [14, 16], further supporting a link between ceramide levels and COVID-19 severity and the observed association between use of FIASMA psychotropic medications and reduced risk of death or intubation among COVID-19 hospitalized patients [6, 9] through potential subsequent antiviral and anti-inflammatory effects.

However, several alternative mechanisms could be proposed to explain this association. First, antiviral effects, i.e. inhibition of viral replication, of FIASMA medications might underlie this relationship, as suggested by prior preclinical studies [8, 51–54]. Second, many SSRIs, except for example paroxetine, and especially fluvoxamine, fluoxetine and escitalopram, have agonist effect on Sigma-1 receptors (S1R) [58, 59], which have been shown to restrict the endonuclease activity of an Endoplasmic Reticulum (ER) stress sensor called Inositol-Requiring Enzyme 1 (IRE1) and to reduce cytokine expression without inhibiting classical inflammatory pathways [28, 60–64]. Because several FIASMA antidepressants are also S1R agonists, this mechanism may have overlapped their inhibition effect on the ASM/ceramide system. However, when examining the association between several FIASMA psychotropic medications with low or no affinity for S1R (i.e., paroxetine, duloxetine, and aripiprazole) [58, 65–67] (versus no FIASMA psychotropic medication) and the primary endpoint, main results remained statistically significant (Supplementary Table 7). Third, this association may be partly mediated by the anti-inflammatory effects of FIASMA psychotropic medications, which could be explained by (i) effects on non-S1R-IRE1 pathways, such as NF-κB, inflammasomes, Toll-like receptor 4 (TLR4), or peroxisome proliferator-activated receptor gamma (PPARγ)), and/or (ii) the inhibition of the ASM in endothelial cells and the immune system, which might be independent of Sigma-1 receptors [9, 64]. First, a recent meta-analysis [62] of studies conducted in individuals with major depressive disorder following antidepressant treatment, mostly including SSRIs, supports that, overall, antidepressants may be associated with decreased plasma levels of 4 of 16 tested inflammatory mediators, including IL-10, TNF-α, and CCL-2, which are associated with COVID-19 severity [68], as well as IL-6, which is highly correlated with disease mortality [68, 69]. This anti-inflammatory effect of the antidepressants fluoxetine and fluvoxamine was also observed in vivo in LPS-induced endotoxic septic shock mouse models [61, 63]. Second, a study from Creeden and colleagues compared differential gene expression signatures from drug-treated cell lines with those from genetic knockdown of select cytokine storm-related inflammatory genes. Interestingly, they found greater concordance in these signatures with the antidepressant fluoxetine than with dexamethasone, a steroid widely used to treat patients with severe COVID-19 [70]. Lastly, prior in vitro and in vivo studies suggest that antipsychotics may induce anti-inflammatory effects dependent on glia activation, and that this activity may not be shared by all antipsychotics. However, this anti-inflammatory effect was observed for both FIASMA antipsychotics (e.g., chlorpromazine) and non-FIASMA ones (e.g., haloperidol and risperidone). Finally, other potential mechanisms may include reduction in platelet aggregation, decreased mast cell degranulation, increased melatonin levels, interference with endolysosomal viral trafficking, and anti-oxidant activities [6, 9, 64]. If the association between FIASMA psychotropic medication use and reduced risk of intubation or death were confirmed in clinical trials, future studies aiming at disentangling these potentially interrelated mechanisms would be needed.

Results of our study also suggest that psychiatric disorders, including psychotic disorders, substance abuse disorders, and mood or anxiety disorders, and classes of psychotropic medications such as antipsychotics, mood stabilizers, or benzodiazepines or Z-drugs, may not be significantly associated with the risk of intubation or death among inpatients with psychiatric disorders and severe COVID-19, following adjustments for age, sex, obesity, other medical comorbidities, and psychotropic medications. Our findings are not in line with those of a meta- analysis by Vai et al. [71], suggesting that pre-existing psychiatric disorders, in particular psychotic and mood disorders, and exposure to antipsychotics and anxiolytics may be associated with COVID-19 mortality. However, a critical limitation for interpreting these findings is that only 9 of 23 studies included in this meta-analysis adjusted for a limited number of comorbid medical conditions. Because comorbid medical illnesses are more prevalent in people with psychiatric disorders [72, 73] than in the general population, and are strongly associated with increased risk of COVID-19-related mortality [74], it is possible that these associations are confounded by medical comorbidities [73, 75]. Therefore, discrepancies between our results and those of Vai et al. [71] may be explained by (i) the choice of a different population in our study, i.e., only inpatients with psychiatric disorders and severe COVID-19, instead of a population with and without psychiatric disorders in the Vai et al. [71] study, (ii) our ability in our analyses to adjust for main medical risk factors of severe COVID-19 (e.g., medical conditions and obesity), (iii) the choice of a partly different outcome (intubation or death versus mortality), and/or (iv) the limited statistical power of our study due to the restricted sample size of 545 adult inpatients with psychiatric disorders and severe COVID-19. Taken together, these results suggest that patients with psychiatric disorders may have increased risk of death due to COVID-19, which could be explained by their greater number of medical risk factors of severe COVID-19 [25]. Future studies taking into account main medical risk factors of severe COVID-19, i.e., age, obesity and medical comorbidities, are needed to determine whether the risk of COVID-19-related mortality is similar or different across psychiatric diagnoses and psychotropic medications prescribed.

Our study has several limitations. First, there are two possible major inherent biases in observational studies: unmeasured confounding and confounding by indication. We tried to minimize the effects of confounding in several different ways. First, we used an analysis with inverse probability weighting to minimize the effects of confounding by indication [43, 44]. Second, we performed multiple sensitivity analyses, which showed similar results. Finally, although some amount of unmeasured confounding may remain, our analyses adjusted for numerous potential confounders. Other limitations include missing data for some baseline characteristic variables (i.e., 11.5%), which might be explained by the overwhelming of all hospital units during the COVID-19 peak incidence, and different results might have been observed during a lower COVID-19 incidence period. However, imputation of missing data did not alter the significance of our results (data available on request). Second, inflation of type I error might have occurred in secondary exploratory analyses due to multiple testing. Third, data on several FIASMA psychotropic medications, such as fluvoxamine or maprotiline, were not available because no patients with psychiatric disorders hospitalized for severe COVID-19 received them at study baseline in AP-HP hospitals. Fourth, sample sizes for studying the associations of individual FIASMA psychotropic medications with the outcome was ≤44 patients, substantially limiting the statistical power of exploratory analyses. Fifth, this study cannot establish a causal relationship between FIASMA psychotropic medication use and reduced risk of intubation or death [76]. Finally, for some of the psychotropic medications administered, we had no experimental data to assign them to FIASMAs or non-FIASMAs, and these substances were assigned to non-FIASMAs. This approach is conservative and may lead to an underestimation of the observed effect. Finally, despite the multicenter design, our results may not be generalizable to outpatients or other regions.

In this multicenter observational retrospective study, FIASMA psychotropic medication use at baseline was significantly associated with a reduced risk of intubation or death among adult patients with psychiatric disorders hospitalized for severe COVID-19. This association was not specific to one FIASMA psychotropic class or medication. Our results also indicate that patients taking a FIASMA antidepressant may have a significantly reduced risk of intubation or death compared with those taking a non-FIASMA antidepressant. These findings suggest the usefulness of the ASM/ceramide system framework in COVID-19. They also support the non-stop of FIASMA psychotropic medications in these patients during SARS-CoV-2 infection. Double-blind controlled randomized clinical trials of these medications against COVID-19 are needed to confirm these results, starting with FIASMA molecules with the highest in vitro inhibition effect on ASM and the most easy to use, including the highest safety margin and tolerability, the better global availability, and the lowest cost to allow their use, if proven effective, also in resource-poor unvaccinated countries [6, 7].

## Supplementary information

Supplemental material

## Data Availability

Data from the AP-HP Health Data Warehouse can be obtained upon request at https://eds.aphp.fr//.
