# Supplementary material for: Association between FIASMA psychotropic medications and reduced risk of intubation or death in individuals with psychiatric disorders hospitalized for severe COVID-19: an observational multicenter study
Source: Transl Psychiatry. 2022 Mar 3;12:90. doi: 10.1038/s41398-022-01804-5 (PMC8892828; doi:10.1038/s41398-022-01804-5)
Supplement: Supplementary file 1 — Supplemental material [file 41398_2022_1804_MOESM1_ESM.pdf]

SUPPLEMENTARY MATERIAL

Supplementary Text

Variable assessment

We obtained the following data for each patient at the time of the hospitalization: sex; age, which was categorized into 4 classes based on the OpenSAFELY study results [1] (i.e. 18-50, 51-70, 71-80, 81+); hospital, which was categorized into 4 classes following the administrative clustering of AP-HP hospitals in Paris and its suburbs based on their geographical location (i.e., AP-HP Centre – Paris University, Henri Mondor University Hospitals and at home hospitalization; AP-HP Nord and Hôpitaux Universitaires Paris Seine-Saint-Denis; AP-HP Paris Saclay University; and AP-HP Sorbonne University); obesity, which was defined as having a body mass index higher than 30 kg/m^2 or an ICD-10 diagnosis code for obesity (E66.0, E66.1, E66.2, E66.8, E66.9); self-reported current smoking status; number of medical conditions associated with severe COVID-19 [2–4], based on ICD-10 diagnosis codes, including diabetes mellitus (E11), diseases of the circulatory system (I00-I99), diseases of the respiratory system (J00-J99), neoplasms (C00-D49), and diseases of the blood and blood-forming organs and certain disorders involving the immune mechanism (D5-D8); and any medication prescribed according to compassionate use or as part of a clinical trial (e.g. hydroxychloroquine, azithromycin, remdesivir, tocilizumab, sarilumab, or dexamethasone). To take into account possible confounding by indication bias for psychotropic medications, we recorded whether patients had any current diagnosis of substance use disorder (F10-F19), psychotic disorder (F20-F29), mood or anxiety disorder (F30-F48), delirium or dementia (F05, R41.0 and G31.0), or any other psychiatric disorder (F01-F04, F06-F09 and F50-F99) based on ICD-10 diagnosis codes, and whether they were prescribed any antidepressant, mood stabilizer, benzodiazepine or Z-drug, or antipsychotic medication.

All medical notes and prescriptions are computerized in Greater Paris University hospitals. Medications including their dosage, frequency, date, and mode of administration were identified from medication administration data or scanned hand-written medical prescriptions, through two deep learning models based on BERT contextual embeddings [5], one for the medications and another for their mode of administration. The model was trained on the APmed corpus [6], a previously annotated dataset for this task. Extracted medications names were then normalized to the Anatomical Therapeutic Chemical (ATC) terminology using approximate string matching.

References

1. Williamson EJ, Walker AJ, Bhaskaran K, Bacon S, Bates C, Morton CE, et al. Factors associated with COVID-19-related death using OpenSAFELY. Nature. 2020;584:430–436.
2. Lagunas-Rangel FA. Neutrophil-to-lymphocyte ratio and lymphocyte-to-C-reactive protein ratio in patients with severe coronavirus disease 2019 (COVID-19): a meta-analysis. Journal of Medical Virology. 2020;92:1733–1734.
3. Zhang C, Wu Z, Li J-W, Zhao H, Wang G-Q. Cytokine release syndrome in severe COVID-19: interleukin-6 receptor antagonist tocilizumab may be the key to reduce mortality. International Journal of Antimicrobial Agents. 2020;55:105954.
4. Geleris J, Sun Y, Platt J, Zucker J, Baldwin M, Hripcsak G, et al. Observational Study of Hydroxychloroquine in Hospitalized Patients with Covid-19. N Engl J Med. 2020;382:2411–2418.
5. Devlin J, Chang M-W, Lee K, Toutanova K. BERT: Pre-training of Deep Bidirectional Transformers for Language Understanding. ArXiv. 2019. 24 May 2019. https://doi.org/1810.04805.
6. Jouffroy J, Feldman SF, Lerner I, Rance B, Neuraz A, Burgun A. MedExt: combining expert knowledge and deep learning for medication extraction from French clinical texts. 2020. 23 January 2020.

Supplementary Table 1. Associations of baseline clinical characteristics with the composite endpoint of intubation or death in the cohort of adult patients with psychiatric disorders who had been admitted to AP-HP hospitals for severe COVID-19.

	Full sample (N=545)	With the endpoint (N=272)	Without the endpoint (N=273)	Crude analysis	Multivariate analysis	Multicollinearity diagnosis
	**N (%)**	**N (%)**	**N (%)**	**HR (95% CI; p-value)**	**HR (95% CI; p-value)**	**GVIF**
Age						1.08
18 to 50 years	45 (8.26%)	15 (33.3%)	30 (66.7%)	Ref.	Ref.	
51 to 70 years	148 (27.2%)	57 (38.5%)	91 (61.5%)	0.98 (0.56 - 1.74; 0.956)	0.86 (0.42 - 1.77; 0.688)	
71 to 80 years	112 (20.6%)	60 (53.6%)	52 (46.4%)	1.53 (0.87 - 2.69; 0.143)	1.48 (0.73 - 2.97; 0.275)	
More than 80 years	240 (44.0%)	140 (58.3%)	100 (41.7%)	1.64 (0.96 - 2.79; 0.069)	1.64 (0.82 - 3.28; 0.161)	
Sex						1.10
Women	250 (45.9%)	110 (44.0%)	140 (56.0%)	Ref.	Ref.	
Men	295 (54.1%)	162 (54.9%)	133 (45.1%)	1.42 (1.11 - 1.81; 0.005*)	1.43 (1.11 - 1.85; 0.006*)	
Hospital						1.04
AP-HP Centre – Paris University, Henri Mondor University Hospitals and at home hospitalization	151 (27.7%)	90 (59.6%)	61 (40.4%)	Ref.	Ref.	
AP-HP Nord and Hôpitaux Universitaires Paris Seine-Saint-Denis	122 (22.4%)	50 (41.0%)	72 (59.0%)	0.65 (0.46 - 0.92; 0.016*)	1.07 (0.73 - 1.55; 0.743)	
AP-HP Paris Saclay University	154 (28.3%)	78 (50.6%)	76 (49.4%)	0.73 (0.54 - 0.98; 0.039*)	0.70 (0.52 - 0.95; 0.022*)	
AP-HP Sorbonne University	118 (21.7%)	54 (45.8%)	64 (54.2%)	0.65 (0.46 - 0.91; 0.013*)	0.73 (0.49 - 1.10; 0.132)	
Obesity [a]						1.05
Yes	119 (21.8%)	60 (50.4%)	59 (49.6%)	1.00 (0.75 - 1.34; 0.987)	1.42 (1.04 - 1.93; 0.026*)	
No	426 (78.2%)	212 (49.8%)	214 (50.2%)	Ref.	Ref.	
Smoking [b]						1.05
Yes	118 (21.7%)	62 (52.5%)	56 (47.5%)	0.87 (0.65 - 1.16; 0.350)	0.83 (0.60 - 1.14; 0.258)	
No	427 (78.3%)	210 (49.2%)	217 (50.8%)	Ref.	Ref.	
Number of medical conditions [c]						1.07
0	93 (17.1%)	18 (19.4%)	75 (80.6%)	Ref.	Ref.	

1	54 (9.91%)	18 (33.3%)	36 (66.7%)	2.17 (1.13 - 4.17; 0.021*)	2.08 (1.06 - 4.06; 0.032*)	
2 or more	398 (73.0%)	236 (59.3%)	162 (40.7%)	4.00 (2.47 - 6.46; <0.001*)	3.43 (1.96 - 6.00; <0.001*)	
Any current substance abuse disorder [d]						1.21
Yes	67 (12.3%)	38 (56.7%)	29 (43.3%)	1.37 (0.97 - 1.92; 0.075)	1.13 (0.71 - 1.79; 0.611)	
No	478 (87.7%)	234 (49.0%)	244 (51.0%)	Ref.	Ref.	
Any current psychotic disorder [e]						1.07
Yes	31 (5.69%)	13 (41.9%)	18 (58.1%)	0.78 (0.44 - 1.35; 0.371)	0.78 (0.41 - 1.50; 0.460)	
No	514 (94.3%)	259 (50.4%)	255 (49.6%)	Ref.	Ref.	
Any current mood or anxiety disorder [f]						1.09
Yes	93 (17.1%)	52 (55.9%)	41 (44.1%)	1.08 (0.80 - 1.46; 0.632)	0.87 (0.63 - 1.19; 0.388)	
No	452 (82.9%)	220 (48.7%)	232 (51.3%)	Ref.	Ref.	
Delirium or dementia [g]						1.18
Yes	153 (28.1%)	99 (64.7%)	54 (35.3%)	1.51 (1.18 - 1.94; 0.001*)	1.04 (0.76 - 1.41; 0.826)	
No	392 (71.9%)	173 (44.1%)	219 (55.9%)	Ref.	Ref.	
Any other psychiatric disorder [h]						1.13
Yes	43 (7.89%)	28 (65.1%)	15 (34.9%)	1.79 (1.21 - 2.65; 0.004*)	1.09 (0.65 - 1.84; 0.734)	
No	502 (92.1%)	244 (48.6%)	258 (51.4%)	Ref.	Ref.	
Medication according to compassionate use or as part of a clinical trial [i]						1.09
Yes	142 (26.1%)	66 (46.5%)	76 (53.5%)	0.88 (0.66 - 1.16; 0.349)	0.88 (0.65 - 1.19; 0.403)	
No	403 (73.9%)	206 (51.1%)	197 (48.9%)	Ref.	Ref.	
Any antidepressant						1.15
Yes	238 (43.7%)	95 (39.9%)	143 (60.1%)	0.53 (0.42 - 0.68; <0.001*)	0.70 (0.53 - 0.92; 0.012*)	
No	307 (56.3%)	177 (57.7%)	130 (42.3%)	Ref.	Ref.	
Any antipsychotic medication						1.09
Yes	93 (17.1%)	30 (32.3%)	63 (67.7%)	0.56 (0.38 - 0.82; 0.003*)	0.65 (0.42 - 1.02; 0.061)	
No	452 (82.9%)	242 (53.5%)	210 (46.5%)	Ref.	Ref.	
Any mood stabilizer medication [j]						1.10
Yes	92 (16.9%)	34 (37.0%)	58 (63.0%)	0.64 (0.45 - 0.92; 0.016*)	0.86 (0.59 - 1.27; 0.454)	
No	453 (83.1%)	238 (52.5%)	215 (47.5%)	Ref.	Ref.	

Any benzodiazepine or Z-drug						1.10
Yes	239 (43.9%)	122 (51.0%)	117 (49.0%)	0.87 (0.68 - 1.11; 0.251)	1.02 (0.77 - 1.35; 0.895)	
No	306 (56.1%)	150 (49.0%)	156 (51.0%)	Ref.	Ref.	

[a] Defined as having a body-mass index higher than 30 kg/m^2 or an International Statistical Classification of Diseases and Related Health Problems (ICD-10) diagnosis code for obesity (E66.0, E66.1, E66.2, E66.8, E66.9).
[b] Current smoking status was self-reported.
[c] Assessed using ICD-10 diagnosis codes for diabetes mellitus (E11), diseases of the circulatory system (I00-I99), diseases of the respiratory system (J00-J99), neoplasms (C00-D49), diseases of the blood and blood-forming organs and certain disorders involving the immune mechanism (D5-D8), frontotemporal dementia (G31.0), peptic ulcer (K27), diseases of liver (K70-K95), hemiplegia or paraplegia (G81-G82), acute kidney failure or chronic kidney disease (N17-N19), and HIV (B20).
[d] Assessed using ICD-10 diagnosis codes for mental and behavioural disorders due to psychoactive substance use (F10-F19).
[e] Assessed using ICD-10 diagnosis codes for psychotic disorders (F20-F29).
[f] Assessed using ICD-10 diagnosis codes for mood and anxiety disorders (F30-F48).
[g] Assessed using ICD-10 diagnosis codes for delirium or dementia (F05, R41.0 and G31.0).
[h] Assessed using ICD-10 diagnosis codes for other psychiatric disorders (F01-F04, F06-F09 and F50-F99).
[i] Any medication prescribed as part of a clinical trial or according to compassionate use (e.g., hydroxychloroquine, azithromycin, remdesivir, tocilizumab, sarilumab, or dexamethasone).
[j] Included lithium or antiepileptic medications with mood stabilizing properties.
* Two-sided p-value is significant (p<0.05).
Abbreviations: HR, hazard ratio, CI confidence interval.

Supplementary Table 2. Backward stepwise regression models.

Step 1	AIC
Delirium or dementia, Any antidepressant , Any benzodiazepine or Z-drug , Any other psychiatric disorder, Any current substance abuse disorder, Medication according to compassionate use or as part of a clinical trial, Any current psychotic disorder, Any mood stabilizer medication, Any antipsychotic, Smoking, Any current mood or anxiety disorder, Hospital, Obesity, Sex, Any FIASMA, Age, Number of conditions	2976.87
**Step 2**	
Delirium or dementia, Any benzodiazepine or Z-drug, Any other psychiatric disorder, Any current substance abuse disorder, Medication according to compassionate use or as part of a clinical trial, Any current psychotic disorder, Any mood stabilizer medication, Any antipsychotic, Smoking, Any current mood or anxiety disorder, Hospital, Obesity, Sex, Any FIASMA, Age, Number of conditions	2974.87
**Step 3**	
Any benzodiazepine or Z-drug, Any other psychiatric disorder, Any current substance abuse disorder, Medication according to compassionate use or as part of a clinical trial, Any current psychotic disorder, Any mood stabilizer medication, Any antipsychotic, Smoking, Any current mood or anxiety disorder, Hospital, Obesity, Sex, Age, Any FIASMA, Number of conditions	2972.88
**Step 4**	
Any other psychiatric disorder, Any current substance abuse disorder, Medication according to compassionate use or as part of a clinical trial, Any current psychotic disorder, Any antipsychotic, Any mood stabilizer medication, Smoking, Any current mood or anxiety disorder, Hospital, Obesity, Sex, Age, Any FIASMA, Number of conditions	2970.91
**Step 5**	
Any current substance abuse disorder, Medication according to compassionate use or as part of a clinical trial, Any antipsychotic, Any current psychotic disorder, Any mood stabilizer medication, Smoking, Any current mood or anxiety disorder, Hospital, Obesity, Sex, Age, Any FIASMA, Number of conditions	2969
**Step 6**	
Medication according to compassionate use or as part of a clinical trial, Any antipsychotic, Any current psychotic disorder, Smoking, Any mood stabilizer medication, Any current mood or anxiety disorder, Hospital, Obesity, Sex, Age, Any FIASMA, Number of conditions	2967.12
**Step 7**	
Any antipsychotic, Any current psychotic disorder, Any mood stabilizer medication, Smoking, Any current mood or anxiety disorder, Hospital, Obesity, Sex, Age, Any FIASMA, Number of conditions	2965.54
**Step 8**	

Smoking, Any mood stabilizer medication, Any antipsychotic, Any current mood or anxiety disorder, Hospital, Obesity, Sex, Age, Any FIASMA, Number of conditions	2964.25
**Step 9**	
Smoking, Any antipsychotic, Any current mood or anxiety disorder, Hospital, Obesity, Sex, Age, Any FIASMA, Number of conditions	2962.95
**Step 10**	
Any antipsychotic, Any current mood or anxiety disorder, Hospital, Obesity, Sex, Age, Any FIASMA, Number of conditions	2961.68
**Step 11**	
Any current mood or anxiety disorder, Hospital, Obesity, Sex, Age, Any FIASMA, Number of conditions	2960.77
**Step 12**	
Hospital, Obesity, Sex, Age, Any FIASMA, Number of conditions	2959.87

Supplementary Table 3. Association of individual FIASMA psychotropic classes and molecules at baseline with the risk of intubation or death among patients with psychiatric disorders hospitalized for severe COVID-19.

	Number of events / Number ofpatients	Crude Cox regression analysis	Multivariable Cox regression analysis [a]	Analysis weighted by inverse-probability-weighting weights [a]	Analysis weighted by inverse-probability-weighting weights [a] adjusted for unbalanced covariates	Number of events / Number of patients in the matched control groups	Univariate Cox regression in a 1:2 ratio matched analytic sample [a]	Cox regression in a 1:2 ratio matched analytic sample[a] adjusted for unbalanced covariates
	N (%)	HR (95% CI; p-value)	HR (95% CI; p-value)	HR (95% CI; p-value)	HR (95% CI; p-value)	N (%)	HR (95% CI; p-value)	HR (95% CI; p-value)
No FIASMA psychotropic medication	215 / 381 (56.4)	Ref.	Ref.	Ref.	Ref.	Ref.	Ref.	Ref.
FIASMA Antidepressants	53 / 148 (35.8)	0.47 (0.35 – 0.63; <0.001*)	0.56 (0.41 – 0.76; <0.001*)	0.53 (0.40 – 0.72; <0.001*)	-	152 / 296 (51.4)	0.60 (0.42 – 0.85; 0.004*)	0.52 (0.38 – 0.71; <0.001*)
Amitriptyline	8 / 20 (40.0)	0.43 (0.21 – 0.88; 0.021*)	0.44 (0.20 – 0.95; 0.036*)	0.76 (0.38 – 1.50; 0.426)	0.70 (0.37 – 1.30; 0.252) [c]	21 / 40 (52.5)	0.52 (0.23 – 1.19; 0.123)	0.55 (0.23 – 1.32; 0.183) [d]
Clomipramine	1 / 4 (25.0)	0.46 (0.07 – 3.30; 0.441)	NA	NA	NA	NA	NA	NA
Duloxetine	4 / 11 (36.4)	0.38 (0.14 – 1.03; 0.056)	NA	NA	NA	NA	NA	NA
Escitalopram	12 / 42 (28.6)	0.35 (0.20 – 0.63; <0.001*)	0.61 (0.37 – 1.01 ; 0.005*)	0.39 (0.21 – 0.70; 0.002*)	0.34 (0.18 – 0.63; <0.001*) [e]	41 / 84 (48.8)	0.42 (0.22 – 0.80; 0.009*)	0.40 (0.21 – 0.76; 0.006*) [f]
Fluoxetine	4 / 14 (28.6)	0.29 (0.11 – 0.79; 0.015*)	NA	NA	NA	NA	NA	NA
Paroxetine	19 / 44 (43.2)	0.54 (0.33 – 0.86 ; 0.010*)	0.65 (0.39 – 1.04 ; 0.007*)	0.85 (0.54 – 1.35; 0.495)	0.77 (0.48 – 1.23; 0.272) [g]	43 / 88 (48.9)	0.63 (0.36 – 1.09 ; 0.097)	0.61 (0.35 – 1.06 ; 0.078) [h]
Sertraline	7 / 21 (33.3)	0.41 (0.19 – 0.86 ; 0.019*)	0.47 (0.24 – 0.93 ; 0.029*)	0.57 (0.28 – 1.18 ; 0.129)	0.50 (0.21 – 1.20 ; 0.119) [i]	15 / 42 (35.7)	0.72 (0.29 – 1.76 ; 0.468)	0.77 (0.31 – 1.89 ; 0.564) [j]
FIASMA Antipsychotics	4 / 13 (30.8)	0.38 (0.14 – 1.03; 0.058)	0.40 (0.17 – 0.97; 0.042*)	0.58 (0.23 – 1.47; 0.249)	0.58 (0.19 – 1.72; 0.324) [k]	12 / 26 (46.2)	0.42 (0.13 – 1.32; 0.139)	0.25 (0.07 - 0.90; 0.034*)
Aripiprazole	1 / 7 (14.3)	0.16 (0.02 – 1.17; 0.071)	NA	NA	NA	NA	NA	NA
Chlorpromazine	3 / 6 (50.0)	0.75 (0.35 – 1.61; 0.457)	NA	NA	NA	NA	NA	NA

FIASMA Antihistaminics							
Hydroxyzine	4 / 17 (23.5)	0.21 (0.08 – 0.58; 0.002*)	0.16 (0.05 – 0.55; 0.004*)	0.17 (0.03 – 0.84; 0.030*)	0.26 (0.09 – 0.72; 0.010*) [l]	18 / 34 (52.9)	0.27 (0.09 – 0.82; 0.020*)	0.17 (0.05 – 0.57; 0.005*) [m]

[a] Adjusted for age, sex, hospital, obesity, and number of medical conditions.
[b] Adjusted for sex, hospital and number of medical conditions.
[c] Adjusted for age, hospital, and number of medical conditions.
[d] Adjusted for sex and hospital.
[e] Adjusted for age and number of medical conditions.
[f] Adjusted for sex and hospital.
[g] Adjusted for sex, hospital, and number of medical conditions.
[h] Adjusted for sex and obesity.
[i] Adjusted for sex and number of medical conditions.
[j] Adjusted for sex and obesity.
[k] Adjusted for age, sex, hospital, obesity, and number of medical conditions.
[l] Adjusted for age, sex, hospital, obesity, and number of medical conditions.
[m] Adjusted for sex and hospital.
* Two-sided p-value is significant (p<0.05).
Abbreviations: HR, hazard ratio, CI confidence interval, NA, not applicable.
Non-FIASMA psychotropic medications include medications that have either no or a relatively low in vitro FIASMA effect corresponding to an in vitro residual ASM activity lower than 50%, or for which the FIASMA effect status is unknown.

Supplementary Table 4. Association between FIASMA and non-FIASMA psychotropic medication use and the endpoint of intubation or death among patients with psychiatric disorders hospitalized for severe COVID-19.

	**Number of events / Number of patients**	**Crude Cox regression analysis**	**Multivariable Cox regression analysis [a]**	**Analysis weighted by inverse-probability-weighting weights [a]**	**Analysis weighted by inverse-probability-weighting weights [a] adjusted for unbalanced covariates**	**Number of events / Number of patients in the matched groups**	**Univariate Cox regression in a 1:2 ratio matched analytic sample [a]**	**Cox regression in a 1:2 ratio matched analytic sample[a] adjusted for unbalanced covariates**
	N (%)	HR (95%CI; p-value)	HR (95%CI; p-value)	HR (95%CI; p-value)	HR (95%CI; p-value)	N (%)	HR (95%CI; p-value)	HR (95%CI; p-value)
FIASMA antidepressants	53 / 148 (35.8)	0.57 (0.38 – 0.86 ; 0.007*)	0.46 (0.29 – 0.73 ; 0.001*)	0.57 (0.37 – 0.87 ; 0.009*)	-	34 / 90 (37.8)	0.61 (0.38 – 0.97 ; 0.036*)	0.60 (0.38 – 0.95 ; 0.030*)
Other antidepressants	42 / 90 (46.7)	Ref.	Ref.	Ref.	Ref.	42 / 90 (46.7)	Ref.	Ref.
FIASMA antipsychotics	4 / 13 (30.8)	0.97 (0.367 – 2.53; 0.947)	NA	NA	NA	4 / 13 (30.8)	0.57 (0.17 – 1.89; 0.356)	NA
Other antipsychotics	12 / 45 (26.7)	Ref.	Ref.	Ref.	NA	5 / 13 (38.5)	Ref.	NA
FIASMA antihistaminics	4 / 17 (23.5)	1.01 (0.11 – 9.13; 0.990)	NA	NA	NA	NA	NA	NA
Other antihistaminics	1 / 5 (20.0)	Ref.	Ref.	Ref.	Ref.	NA	Ref.	Ref.

[a] Adjusted for age, sex, hospital, obesity, and number of medical conditions.
[b] Adjusted for hospital.
* Two-sided p-value is significant (p<0.05).
Abbreviations: HR, hazard ratio, CI confidence interval, NA, not applicable.
Other psychotropic medications include medications that have either no or a relatively low *in vitro* FIASMA effect corresponding to an *in vitro* residual ASM activity lower than 50%, or for which the FIASMA effect status is unknown.

Supplementary Table 5. Association between FIASMA psychotropic medication use at baseline and intubation or death in patients with psychiatric disorders hospitalized for COVID-19 with and without clinical severity of COVID-19 at admission (N=1,406).

	**Number of events / Number of patients**	**Crude Cox regression analysis**	**Multivariable Cox regression analysis [a]**	**Analysis weighted by inverse-probability-weighting weights**	**Analysis weighted by inverse-probability-weighting weights [a] adjusted for unbalanced covariates**	**Number of events / Number of patients in the matched groups**	**Univariate Cox regression in a 1:1 ratio matched analytic sample [a]**	**Cox regression in a 1:1 ratio matched analytic sample [a] adjusted for unbalanced covariates**
	N (%)	HR (95% CI; p-value)	HR (95% CI; p-value)	HR (95% CI; p-value)	HR (95% CI; p-value)	N (%)	HR (95% CI; p-value)	HR (95% CI; p-value)
No FIASMA psychotropic medication	15 / 902 (38.9)	Ref.	Ref.	Ref.	Ref.	161 / 504 (31.9)	Ref.	Ref.
Any FIASMA psychotropic medication	351 / 504 (22.6)	0.47 (0.38 – 0.58; <0.001*)	0.67 (0.52 – 0.87; <0.001*)	0.63 (0.51 – 0.78; <0.001*)	NA	114 / 504 (22.6)	0.60 (0.48 – 0.77; <0.001*)	0.64 (0.51 – 0.82; <0.001*) [b]

[a] Adjusted for age, sex, hospital, obesity and number of medical conditions.
[b] Additionally adjusted for sex and number of medical conditions.
* Two-sided p-value is significant (p<0.05).
Abbreviations: HR, hazard ratio, CI confidence interval.
Non-FIASMA psychotropic medications include medications that have either no or a relatively low in vitro FIASMA effect corresponding to an in vitro residual ASM activity lower than 50%, or for which the FIASMA effect status is unknown.

Supplementary Table 6. Association between FIASMA psychotropic medication use at baseline and risk of intubation or death among patients with psychiatric disorders hospitalized for severe COVID-19 while considering venlafaxine, mirtazapine and citalopram as FIASMA antidepressants.

	Number of events / Number of patients	Crude Cox regression analysis	Multivariable Cox regression analysis [a]	Analysis weighted by inverse-probability-weighting weights	Analysis weighted by inverse-probability-weighting weights [a] adjusted for unbalanced covariates [b]	Number of events / Number of patients in the matched groups	Univariate Cox regression in a 1:2 ratio matched analytic sample [a]	Cox regression in a 1:2 ratio matched analytic sample [a] adjusted for unbalanced covariates [c]
	N (%)	HR (95% CI; p-value)	HR (95% CI; p-value)	HR (95% CI; p-value)	HR (95% CI; p-value)	N (%)	HR (95% CI; p-value)	HR (95% CI; p-value)
No FIASMA psychotropic medication	200 / 354 (56.5)	Ref.	Ref.	Ref.	Ref.	92 / 191 (48.2)	Ref.	Ref.
Any FIASMA psychotropic medication	72 / 191 (37.7)	0.48 (0.37 – 0.63; <0.001*)	0.55 (0.42 – 0.73; <0.001*)	0.61 (0.46 – 0.80; <0.001*)	0.54 (0.41 – 0.71; <0.001*)	72 / 191 (37.7)	0.59 (0.43 – 0.80; 0.001*)	0.58 (0.43 – 0.80; 0.001*)

[a] Adjusted for age, sex, hospital, obesity and number of medical conditions.
[b] Additionally adjusted for sex, and number of medical conditions.
[c] Additionally adjusted for sex, and obesity.
* Two-sided p-value is significant (p<0.05).
Abbreviations: HR, hazard ratio, CI confidence interval.
Non-FIASMA psychotropic medications include medications that have either no or a relatively low in vitro FIASMA effect corresponding to an in vitro residual ASM activity lower than 50%, or for which the FIASMA effect status is unknown.

Supplementary Table 7. Association between FIASMA medications with low or no affinity for Sigma-1-receptors (i.e., paroxetine, duloxetine, aripiprazole) and the composite endpoint of intubation or death among adult patients with psychiatric disorders hospitalized for severe COVID-19.

	Number ofevents / Number ofpatients	Crude Cox regressio nanalysis	Multivariable Cox regression analysis [a]	Analysis weightedby inverse-probability-weighting weights	Analysis weighted by inverse-probability-weighting weights [a] adjusted for unbalanced covariates [b]	Number of events / Number of patients in the matched groups	Univariate Cox regression in a 1:2 ratio matched analytic sample [a]	Cox regression in a 1:2 ratio matched analytic sample [a] adjusted for unbalanced covariates [c]
	N (%)	HR (95% CI; p-value)	HR (95% CI; p-value)	HR (95% CI; p-value)	HR (95% CI; p-value)	N (%)	HR (95% CI; p-value)	HR (95% CI; p-value)
No FIASMA psychotropic medication	215 / 381 (56.4)	Ref.	Ref.	Ref.	Ref.	30 / 60 (50.0)	Ref.	Ref.
Any FIASMA medication with low or no affinity for Sigma-1-receptors	24 / 60 (40.0)	0.48 (0.31 - 0.73; 0.001*)	0.50 (0.30 - 0.82; 0.007*)	0.73 (0.48 - 1.12; 0.150)	0.62 (0.41 - 0.93; 0.022*)	24 / 60 (40.0)	0.57 (0.33 - 0.98; 0.042*)	0.57 (0.33 - 0.98; 0.041*)

[a] Adjusted for age, sex, hospital, obesity and number of medical conditions.
[b] Additionally adjusted for age, hospital, and number of medical conditions.
[c] Additionally adjusted for sex.
* Two-sided p-value is significant (p<0.05).
Abbreviations: HR, hazard ratio, CI confidence interval.
Non-FIASMA psychotropic medications include medications that have either no or a relatively low in vitro FIASMA effect corresponding to an in vitro residual ASM activity lower than 50%, or for which the FIASMA effect status is unknown.
